# The diagnostic value of multiparameter cardiovascular magnetic resonance for early detection of light-chain amyloidosis from hypertrophic cardiomyopathy patients

**DOI:** 10.3389/fcvm.2022.1017097

**Published:** 2022-10-18

**Authors:** Xiuzheng Yue, Lili Yang, Rui Wang, Queenie Chan, Yanbing Yang, Xiaohong Wu, Xiaowei Ruan, Zhen Zhang, Yuping Wei, Fang Wang

**Affiliations:** ^1^Philips Healthcare, Beijing, China; ^2^Medical Imaging Center, People’s Hospital of Ningxia Hui Autonomous Region (The North University of Nationalities Teaching Hospital), Yinchuan, China; ^3^Philips Healthcare, Hong Kong, Hong Kong SAR, China; ^4^Department of Hematology, People’s Hospital of Ningxia Hui Autonomous Region (The North University of Nationalities Teaching Hospital), Yinchuan, China

**Keywords:** cardiomyopathies cardiac, cardiac amyloidosis (CA), LGE CMR, strain, T1 mapping MRI, ECV

## Abstract

**Background:**

Early-stage amyloidosis of the heart is prone to be underdiagnosed or misdiagnosed, increasing the risk of early heart failure and even death of the patient. To ensure timely intervention for cardiac light-chain amyloidosis (AL CA), it is vital to develop an effective tool for early identification of the disease. Recently, multiparameter cardiovascular magnetic resonance (CMR) has been used as a comprehensive tool to assess myocardial tissue characterization. We aimed to investigate the difference in left ventricular (LV) strain, native T1, extracellular volume (ECV), and late gadolinium enhancement (LGE) between AL CA patients, hypertrophic cardiomyopathy patients (HCM), and healthy control subjects (HA). Moreover, we explored the value of multiparameter CMR for differential diagnosis of the early-stage AL CA patients from HCM patients, who shared similar imaging characteristics under LGE imaging.

**Methods:**

A total of 38 AL CA patients, 16 HCM patients, and 17 HA people were prospectively recruited. All subjects underwent LGE imaging, Cine images, and T1 mapping on a 3T scanner. The LV LGE pattern was recorded as none, patchy or global. LV strain, native T1, and ECV were measured semi-automatically using dedicated CMR software. According to clinical and biochemical markers, all patients were classified as Mayo stage I/II and Mayo stage IIIa/IIIb. Univariable and multivariable logistic regression models were utilized to identify independent predictors of early-stage AL CA from HCM patients. Receiver operator characteristic (ROC) curve analysis and Youden’s test were done to determine the accuracy of multiparameter CMR in diagnosing Mayo stage I/II AL CA and establish a cut-off value.

**Results:**

For Mayo stage I/II AL CA patients, the global longitudinal strain (GLS) absolute value (11.9 ± 3.0 vs. 9.5 ± 1.8, *P* < 0.001) and the global circumferential strain (GCS) absolute value (19.0 ± 3.6 vs. 9.5 ± 1.8, *P* < 0.001) were significantly higher than in HCM patients. The native T1 (1334.9 ± 49.9 vs. 1318.2 ± 32.4 ms, *P* < 0.0001) and ECV values (37.8 ± 5.7 vs. 31.3 ± 2.5%, *P* < 0.0001) were higher than that of HCM patients. In multiparameter CMR models, GCS (2.097, 95% CI: 1.292–3.403, *P* = 0.003), GLS (1.468, 95% CI: 1.078–1.998, *P* = 0.015), and ECV (0.727, 95% CI: 0.569–0.929, *P* = 0.011) were the significant variables for the discrimination of the early-stage AL CA patients from HCM patients. ROC curve analysis and Youden’s test were used on GCS, GLS, ECV, and pairwise parameters for differentiating between Mayo stage I/II AL CA and HCM patients, respectively. The combination of GLS, GCS, and ECV mapping could distinguish Mayo stage I/II AL amyloidosis patients from hypertrophic cardiomyopathy with excellent performance (AUC = 0.969, Youden index = 0.813).

**Conclusion:**

In early-stage AL CA patients with atypical LGE, who had similar imaging features as HCM patients, ECV mapping, GCS, and GLS were correlated with the clinical classification of the patients. The combination of GCS, GLS, and ECV could differentiate early-stage AL CA from HCM patients. Multiparameter CMR has the potential to provide an effective and quantitative tool for the early diagnosis of myocardial amyloidosis.

## Background

Primary cardiac light chain amyloidosis (AL CA), the most common form of systemic amyloid disease, is characterized by the extracellular deposition of misfolded proteins overproduced plasma cell dyscrasia (1). Early-stage amyloidosis of the heart may be underdiagnosed and misdiagnosed (2, 3) which is a significant factor in patient mortality. The current clinical approach to identifying AL CA follows a multimodality imaging strategy, which includes echocardiography, cardiac magnetic resonance (CMR), and nuclear imaging (4). Early-stage AL CA patients and hypertrophic cardiomyopathy (HCM) patients share similar CMR imaging features such as the absence of patch LGE pattern, elevated left ventricular (LV) wall thickness, etc. This similarity brings difficulty in distinguishing early-stage AL CA from HCM. Hence, it is crucial to develop a feasible and non-invasive tool to discriminate against early-stage AL CA patients without typical LGE patterns in clinical practices.

Cardiovascular magnetic resonance could provide high resolution, robust functional assessment, and superior tissue characterization for the diagnosis of AL CA. In several publications, it had been demonstrated that typical AL CA patients had thickened LV walls associated with global, diffuse subendocardial late gadolinium enhancement (LGE) pattern (5, 6). A systematic review with meta-analysis (5 studies, *n* = 257) demonstrated a pooled sensitivity and specificity of 85 and 92% for the diagnosis of cardiac amyloidosis by LGE (7). Recently, myocardial feature tracking CMR and quantitative CMR T1 mapping methods have attracted more attention for the detection and differentiation of AL CA. Previous studies have demonstrated the potential of native T1 and extracellular volume (ECV) values combined with hematocrit measurements in detecting and differentiating CA. ECV can be used for predicting mortality for AL CA patients during short-term follow-up (8–10). The strain analysis based on cine image has emerged as a feasible tool for assessing the myocardial deformation in AL CA patients, and global longitudinal strain (GLS) could be the new promising indicator, even in patients without LV LGE or abnormal ejection fraction (EF) (11, 12).

Cardiovascular magnetic resonance has proven to be an important tool for assessing cardiac amyloidosis since it offers a variety of imaging versatility and dimensions (10, 11, 13). Recent advances have focused on tracking disease progression and monitoring response to therapy. However, previous CMR studies have predominantly included patients at later stage of AL CA. The early-stage AL CA is still prone to be underdiagnosed or misdiagnosed, leading to early heart failure or death. Therefore, effective identification of early-stage AL CA, especially with atypical LGE patterns, is critical for clinical practice to ensure timely treatment for the disease. Until now, only one case report has demonstrated that using CMR in conjunction with other modalities is valid for the early detection of cardiac transthyretin (ATTR) in carriers of transthyretin mutation (14) rather than AL CA. To the best of our knowledge, there has been no study using multiparameter CMR for the early detection of AL CA published so far. In the present study, we examined a Chinese population with different stages of AL amyloidosis using a 3T scanner with T1 mapping and cine sequences and compared their early diagnostic value of T1 mapping, ECV, and strain with LGE.

## Materials and methods

### Study subjects

This research was approved by the ethics committee and Institutional Review Board of the People’s Hospital of Ningxia Hui Autonomous Region (Institutional Review Board No. 2020-LL-022). All subjects have consented to participate in this study. AL CA patients who were referred for CMR imaging at the People’s Hospital of Ningxia Hui Autonomous Region between 1 January 2019, and 1 March 2022, were included in the study. Approximately 20% of the patients with contraindications either to CMR imaging (i.e., CMR-incompatible devices) or contrast administration (i.e., estimated glomerular filtration rate < 30 ml/min) were excluded.

**FIGURE 1:**
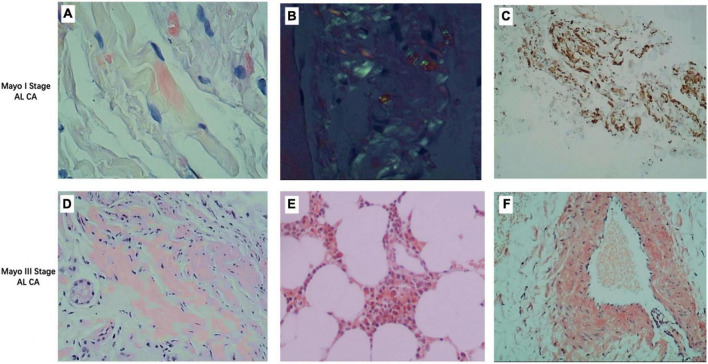
Representative images of different stages of light chain amyloidosis patients. **(A)** Congo red stain fluorescence staining of deltoid muscle of a Mayo I stage patient showing red fluorescent bright of amyloid deposit (×400). **(B)** Congo red stain birefringence of **(A)** under polarized light showing amyloid (×400). **(C)** Immunohistochemistry stains for lambda (+) and kappa (–) light chains in the deltoid muscle of a Mayo I stage patient show amyloid deposits (×400). **(D)** Congo red stain fluorescence staining of the liver of a Mayo III stage patient showing red fluorescent bright of amyloid deposit (×400). **(E)** Congo red stain fluorescence staining of bone marrow stroma of a Mayo III stage patient showing red fluorescent bright of amyloid deposit (×400). **(F)** Congo red stain fluorescence staining of the vascular wall of a Mayo III stage patient showing red fluorescent bright of amyloid deposit (×400).

**FIGURE 2:**
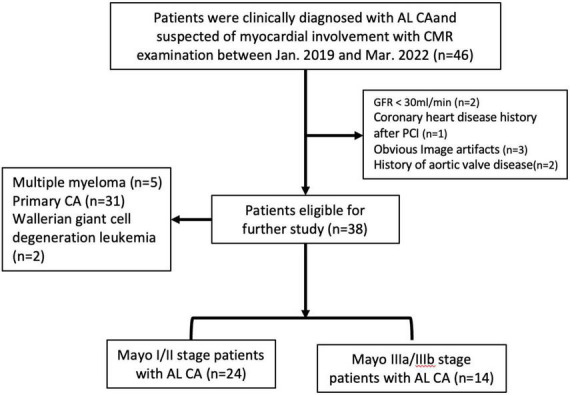
The flowchart of light chain amyloidosis patient selection.

Thirty-eight AL CA patients (61.9 ± 8.4 years; 28 male) were consecutively recruited (Figure 1). All patients had biopsy evidence of light-chain amyloidosis (AL) with positive Congo red stain and light chain deposition confirmed by immunohistochemistry. Representative images are given in Figure 2. The assays were performed in the tissues: skin and fat (*n* = 16), labial gland (*n* = 11), liver (*n* = 6), salivary gland (*n* = 4), muscle (*n* = 4), kidney (*n* = 4), peripheral nerve (*n* = 4), and bone marrow (*n* = 4). All patients underwent laboratory examination of the cardiac biomarkers Troponin I (cTnI, cut-off = 0.1 ng/ml) and N-terminal pro-B-type natriuretic peptide (NT-proBNP, cut-off = 332 ng/L) at baseline and were categorized into three groups based on a four-stage system proposed by the European collaborative studies, which uses high NT-proBNP (NT-proBNP, cut-off = 8,500 ng/L) levels to identify high-risk patients (Mayo2004/European) (15–17): Stage I: both variables below cut-offs; Stage II: one variable above the cut-off; Stage IIIa: both variables above the cut-offs and NT-proBNP below the cut-off (8,500 ng/L); Stage IIIb: both variables above the cut-offs and NT-proBNP above the cut-off (8,500 ng/L). The reason for choosing this four-stage system is its ability to assess eligibility for clinical practices. It is powerful, simple (based on only two markers), and less influenced by confounding factors (18). A hematologist (YW, 8 years) was blinded to the results of CMR imaging and recorded the results of Mayo stage. Sixteen HCM patients (49.2 ± 13.7, 14 males) and seventeen healthy volunteer subjects (HA) (57.4 ± 7.3 years; 10 male, *P* = 0.212). Inclusion criteria for HCM group: patients with a clear family history or genetic testing confirmed hypertrophic cardiomyopathy, CMR showed typical asymmetric thickening of the ventricular septum, normal cardiac function, and normal or positive LGE. Exclusion criteria: ischemic heart disease, structural heart disease, valvular heart disease, and other cardiomyopathy causing myocardial hypertrophy (such as metabolic cardiomyopathy, Fabry disease, etc.). Inclusion criteria for HA group: neither history nor symptoms of cardiovascular disease, negative CMR examination, age between 57 and 70 years. The HCM groups and healthy control group (HA) with all CMR imaging results were recruited.

### Cardiovascular magnetic resonance scanning protocol

Cardiovascular magnetic resonance was performed on a 3.0 Tesla whole-body scanner (Ingenia CX, Philips Healthcare, Best, Netherlands) equipped with a 16-element phased-array torso-cardiac coil. The system can operate at a maximum slew rate of 200 mT/m/ms and a maximum gradient strength of 80 mT/m. A four-lead vector cardiogram was used for electrocardiogram (ECG) gating. To assess left ventricular (LV) myocardial function and mass, 10 consecutive 8 mm short-axis images and 2-, 3-, and 4-chamber long-axis images of the LV were acquired using a cine balanced steady-state free precession sequence (bSSFP). Image parameters were: field of view (FOV): 350 mm × 322 mm, slice thickness 8 mm, slice gap 1 mm, flip angle (FA) 45°, repetition time (TR): 2.2 ms, echo time (TE) 1.08 ms, matrix 196 × 173 resulting in a resolution of 1.79 × 1.87 mm, SENSE factor 1.5, trigger delay end-diastole, native and 15–20 min post-contrast T1 mapping were acquired using a modified look-locker inversion-recovery (MOLLI) images in identical imaging locations, including three short-axis slices (apex, mid-ventricular, and basic) (19). Acquisition schemes 5(3)3 and 4(1)3(1)2 were used for pre-contrast and post-contrast T1 mapping, respectively. The key parameters were as follows: TR/TE: 3.3/1.43 ms; FA 20°, FOV 172 × 150 mm, pixel size 1.42 × 2.6 mm.

Then, a total dose of 0.3 ml/kg gadodiamide (OMNISCAN, GE Healthcare AS) was injected in a 2-phase protocol: 8 ml gadodiamide was infused as a bolus pushed by a 20 ml saline at 4 ml/s. A total of 90 s later, the remaining contrast doses followed by 20 ml saline were infused at a slower rate of 2 ml/s. LGE images were collected ten minutes after contrast injection using a 2D phase-sensitive inversion-recovery (PSIR) gradient-echo pulse sequence with multiple breath-hold. The sequence parameters were as follows: TR/TE/FA, 5.2 ms/1.96 ms/20°; voxel size, 1.4 × 1.4 × 8.0 mm.

### Cardiovascular magnetic resonance image analysis

Two experienced radiologists (FW with 5 years of CMR experience; YY with 3 years of CMR experience) independently analyzed CMR images blinded to clinical data. The LV LGE pattern was classified into three categories according to Boynton et al. (20) and Fontana et al. (21): no LGE, when there were no areas of LGE; patchy LGE, when there were discrete areas of LGE, or there were diffuse areas of LGE in less than half of the short axis images; global LGE, when there was diffuse, transmural LGE in more than half of the short axis images. To exclude artifacts, the LGE was deemed present only if visible in two orthogonal views. Discrepancies were resolved in the consensus during a joint evaluation with a third radiologist (10 years of experience).

Cardiac structure, function, and myocardial deformation were measured semi-automatically using dedicated CMR software (cvi42, version 5.3, Circle Cardiovascular Imaging, Calgary, AB, Canada). The endocardium and epicardium of the left ventricle were automatically contoured on all phases and were checked and corrected manually when needed. Standard parameters of cardiac structure (i.e., inter-ventricular septum thickness, ventricle volume, LV mass, and left atrium area with indexing for body surface area) and EF were measured by contouring the endocardial, epicardial borders on long-axis and short-axis cine images at end-systolic and end-diastolic phase (22). The contouring was checked and corrected manually when needed. Left ventricular maximum wall thickness (LVMWT) was measured at end-diastole in the short-axis orientation of Cine images. Myocardial deformations in longitudinal, radial, and circumferential directions were measured by semi-automatically contouring the endocardial and epicardial borders on 2-, 3-, and 4-chamber long-axis and short-axis cine images at the end-diastolic phase. Three directions of global peak strain were recorded for further analysis. Native T1 and ECV of the 16 American Heart Association (AHA) segments and global LV were measured by semi-automatically contouring the endocardium and epicardium on pre-contrast and post-contrast T1 mapping images combined with the index of hematocrit. LV native T1 and ECV were used for further analysis. The average values of three different global strains, native T1, and ECV measured by the two radiologists were used.

### Statistical analysis

The data were summarized and analyzed using SPSS Statistics (version 26.0 International Business Machines, Inc., Armonk, NY, USA) and R programming language for statistical computing (version 4.2.0, the R Foundation for Statistical Computing). R package [“ggstatsplot,” 0.9.1.9000 (23) and “ggcor” (24)] were used. Continuous variables were expressed as mean ± SD or medians with complete and interquartile ranges (25th to 75th percentile) depending upon the normality of the data. Data were tested for normality using the Shapiro–Wilk test. ANOVA with Bonferroni correction was used for normally distributed data and the Mann–Whitney U test for non-normally distributed data comparison between the groups. Correlation between continuous variables, such as native T1 mapping, GLS, etc., or categorical variables, such as Mayo stage, were assessed using Pearson’s correlation or Spearman *P* correlation. The agreement in CMR parameters between two observers was considered using the interclass correlation coefficient (ICC). Univariable and multivariable logistic regression models were utilized to identify independent predictors of early-stage AL CA from HCM patients. Receiver operator characteristic (ROC) curve analysis and Youden’s test were done to determine the accuracy of multiparameter CMR in diagnosing Mayo stage I/II of AL CA patients and establish a cut-off value. The Power analysis was used to demonstrate that the sample size of HA, CA patients, and HCM patients in this experiment can meet the statistical difference. All tests were two-sided, and *P*-values of <0.05 were considered statistically significant.

## Results

### Study population and clinical stages

Table 1 showed the characteristics of AL CA patients, HCM patients, and healthy controls at baseline. All continuous variables, except for cTnI and NT-proBNP, were normally distributed (Shapiro–Wilk test) and presented as the mean ± SD. cTnI and NT-proBNP were presented as medians (quartiles 1 to quartiles 3). Sixteen HCM patients (14 males) and 38 AL CA patients (28 males) were included in this study. There were 20 (52.6%), 4 (10.6%), and 14 (36.8%) AL CA patients in Mayo stage I (M I), Mayo stage II (M II), and Mayo stage IIIa/IIIb (M III), respectively. Among 16 HCM patients, 5 (31%) patients had hypertension. Among the 24 Mayo stage I/II patients, 16 (67%) patients had hypertension, 4 (17%) patients had diabetes, 6 (25%) patients had coronary heart disease, and 2 (8%) patients had cardiovascular symptoms on admission; among the 14 Mayo stage IIIa/IIIb patients, there was no hypertension, 1 (7%) patient had diabetes, and 3 (21.4%) patients had coronary artery disease, and 7 (50%) patients had cardiovascular-related symptoms on admission. AL CA patients showed lower cTnI [0.038 (0.005–0.055) vs. 0.062 (0.008–0.117), *P* = 0.02] and higher NT-proBNP [1,758 (45–2,755) vs. 525 (75–847), *P* < 0.001] compared to HCM patients. In HCM patients, there were 1 (6.25%), and 15 (93.75%) with no LGE and patchy LGE, respectively. In CA patients, there were 10 (26.3%), 12 (31.6%), and 16 (42.1%) patients with no LGE, patchy LGE, and global LGE, respectively.

**TABLE 1:** Baseline characteristics of the healthy control, HCM patients, and AL CA patients.

Characteristics	HA (*n* = 17)	HCM (*n* = 16)	AL CA patients (*n* = 38)	*P* (HA vs. HCM\HA vs. CA\HCM vs. CA)
Male/female	9¥8	14/2	28¥10	–
Age (years)	58.4 ± 7.3	49.2 ± 13.7	61.9 ± 8.4	0.017*¥0.389¥0.016*
Mayo stage (I/II/IIIa + IIIb)	–	–	20/4/14	–
cTnI (μg/L)	0.000 (0.000–0.001)	0.062 (0.008–0.117)	0.038 (0.005–0.055)	<0.001**¥<0.001**¥0.02*
NT-proBNP (g/ml)	0 (0–8)	525 (75–847)	1,758 (45–2,755)	<0.001**¥<0.001**¥<0.001**
HTN¥DM¥CHD	–	5/0/0	18/6/8	–
**CMR**				
LV EF (%)	62.2 ± 5.8	71.4 ± 8.0	64.2 ± 11.0	0.014*¥0.203¥0.152
Index LVEDV (ml/m^2^)	74.2 ± 9.6	64.6 ± 14.3	66.6 ± 21.3	0.001*¥0.057¥0.216
Index LVESV (ml/m^2^)	28.6 ± 6.9	19.0 ± 8.8	26.8 ± 15.0	0.045*¥0.551¥0.138
LV MI (g/m^2^)	37.4 ± 5.5	72.7 ± 22.5	59.4 ± 16.5	0.001**¥0.004**¥0.359
LVMWT (mm)	6.1 ± 0.7	18.8 ± 3.3	14.9 ± 2.7	<0.001**¥<0.001**¥0.297
LVLGE (none/patchy/extensive)	–	1/15/0	10/12/16	–
LV GRS (%)	45.2 ± 9.4	38.3 ± 12.0	32.8 ± 11.7	0.563¥0.405¥0.891
LV GCS (%)	−21.2 ± 1.8	−15.0 ± 3.2	−18.0 ± 3.3	<0.001**¥0.057¥0.567
LVGLS (%)	−16.8 ± 1.2	−9.5 ± 1.8	−11.2 ± 3.3	<0.001**¥<0.001**¥<0.001**
Native T1 (ms)	1,243.5 ± 29.5	1,318.2 ± 32.4	1,374.6 ± 79.4	<0.001**¥<0.001**¥0.006*
ECV (%)	30.1 ± 2.5	31.3 ± 3.4	42.8 ± 11.5	<0.001**¥<0.001**¥<0.001**

All continuous variables are presented as mean ± SD, except for cTnI, NT-proBNP, which are presented as medians (quartiles 1 to quartiles 3). *P < 0.05, **P < 0.001, statistically significant difference. AL CA, cardiac light-chain amyloidosis; HCM, hypertrophic cardiomyopathy patients; HA, healthy control subjects; cTnI, Cardiac Troponin I; NT-proBNP, N-terminal pro-B-type natriuretic peptide; HTN, hypertension; CHD, coronary artery heart disease; DM, diabetes mellitus; MR, magnetic resonance; LVEF, left ventricle ejection fraction; LVEDV, left ventricle end-diastolic volume index; LVESV, left ventricle end-systolic volume index; LV MI, left ventricle mass index; LGMWT, left ventricle maximum wall thickness; LGE, late gadolinium enhancement; GCS, global circumferential strain; GLS, global longitudinal strain; GRS, global radial strain; ECV, extracellular volume.

### Baseline characteristics of health control, hypertrophic cardiomyopathy patients, and cardiac light-chain amyloidosis patients

All continuous variables were shown in Tables 1, 2, except for cTnI and NT-proBNP, were normally distributed (Shapiro–Wilk test) and presented as the mean ± SD. cTnI and NT-proBNP were presented as medians (quartiles 1 to quartiles 3). Compared to healthy controls, AL CA patients had apparent higher left ventricle mass index (LV MI) (59.4 ± 16.5 vs. 37.4 ± 5.5 g/m^2^, *P* < 0.001) and left maximum wall thickness (LVMWT) (14.9 + 2.7 vs. 6.1 ± 0.7 mm, *P* < 0.001), as well as moderately lower left ventricular end-diastolic volume index (LVEDV) and left ventricle end-systolic volume index (LVESV). Compared with HCM patients, AL CA patients had lower LV MI (59.4 ± 16.5 vs. 72.7 ± 22.5 g/m^2^, *P* = 0.359) and LVMWT (14.9 + 2.7 vs. 18.8 ± 3.3 mm, *P* = 0.297). Among all AL CA patients, Mayo stage I/II AL CA patients had less LV MI (54.2 ± 17.1 vs. 72.7 ± 22.5 g/m^2^, *P* < 0.05) and LVMWT (14.3 ± 2.9 vs. 18.8 ± 3.3 mm, *P* < 0.05) than that of HCM patients. There was no significant difference in the left ventricle ejection fraction (LVEF), the left ventricular end-diastolic volume index (LVESV), and the left ventricular end-systolic volume index (LVESV) between AL CA patients and HCM patients.

**TABLE 2:** Clinical and CMR parameters correlation with hypertrophic cardiomyopathy patient and clinical stage in AL amyloidosis patients.

	HCM	AL CA		Correlation	
	(*n* = 16)	I/II (*n* = 24)	IIIa/IIIb (*n* = 14)	ρ or *r*	*P*
cTnI (ng/L)	0.062 (0.008–0.117)	0.045 (0.038–0.056)^#^	0.060 (0.045–0.106)	0.64	<0.0001**
NT-proBNP (ng/ml)	525 (75–847)	1,949 (215–5,418)^#^	3,575 (767–7,585)	0.82	<0.0001**
**CMR**					
LV EF (%)	71.4 ± 8.0	66.2 ± 11.0	60.9 ± 10.4	−0.23	0.786
LV EDVI (ml/m^2^)	64.8 ± 14.8	71.2 ± 20.1	58.6 ± 21.5	−0.39	<0.05
LVESVI (ml/m^2^)	19.0 ± 8.8	26.4 ± 15.7	27.6 ± 14.1	0.06	0.216
LV MI (g/m^2^)	72.7 ± 22.5	54.2 ± 17.1^#^	68.3 ± 10.8	0.61	<0.0001**
LVMWT (mm)	18.8 ± 3.3	14.3 ± 2.9^#^	15.9 ± 2.3	0.64	<0.0001**
LVLGE (none/patchy/extensive)	1/15/0	10/12/16			
LV GRS (%)	38.3 ± 12.0	36.5 ± 10.9	26.4 ± 10.7	−0.76	<0.0001**
LVGCS (%)	−15.0 ± 3.2	−19.0 ± 3.6^#^	−16.2 ± 1.7	0.64	<0.0001**
LVGLS (%)	−9.5 ± 1.8	−11.9 ± 3.0^#^	−9.9 ± 3.5	0.70	<0.0001**
Native T1 (ms)	1,318.2 ± 32.4	1,334.9 ± 49.9	1,442.7 ± 74.9	0.93	<0.0001**
ECV (%)	31.3 ± 3.4	37.8 ± 5.7^#^	54.1 ± 9.9	0.93	<0.0001**

All continuous variables are presented as mean ± SD, except for cTnI, NT-proBNP, which are presented as medians (quartiles 1 to quartiles 3). Compared between Mayo I¥II and the HCM patients, ^#^*P* < 0.05. **P < 0.001, statistically significant difference. AL CA, cardiac light-chain amyloidosis; HCM, hypertrophic cardiomyopathy patients; HA, healthy control subjects; cTnI, Cardiac Troponin I; NT-proBNP, N-terminal pro-B-type natriuretic peptide; MR, magnetic resonance; LVEF, left ventricle ejection fraction; LV EDV, left ventricle end-diastolic volume index; LVESV, left ventricle end-systolic volume index; LVMI, left ventricle mess index; LVMWT, left ventricle maximum wall thickness; LGE, late gadolinium enhancement; GCS, global circumferential strain; GLS, global longitudinal strain; GRS, global radial strain;, ECV, extracellular volume.

### Cardiovascular magnetic resonance structural and functional parameters

**FIGURE 3:**
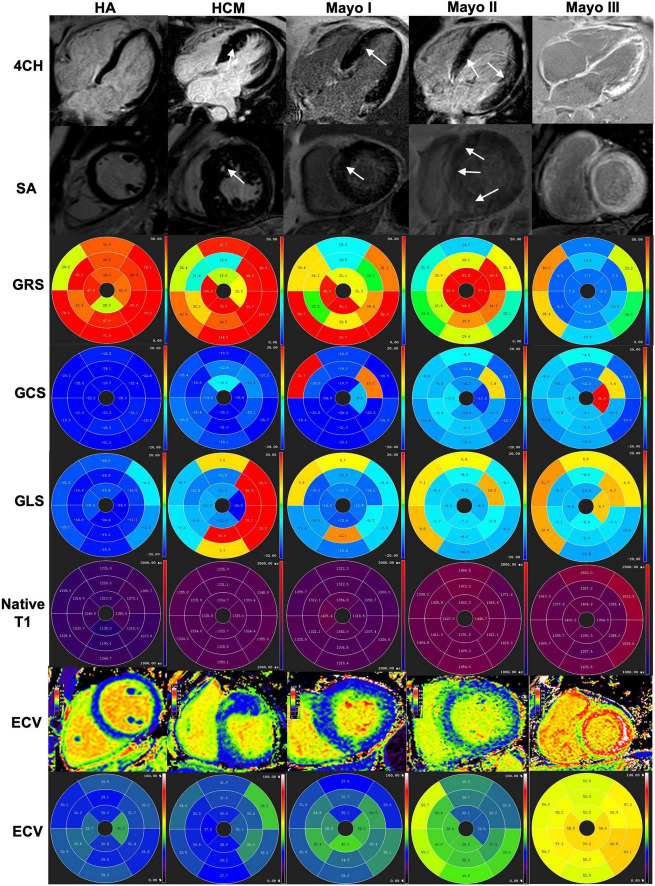
A healthy control subject, an HCM patient and three different stages of AL amyloid patients were shown from left to right and index top to bottom (4CH, SA, GRS, GCS, GLS, native T1, ECV, and ECV bullseye, respectively. **(Column 1)** A healthy control subject without negative LGE and GRS (47.55%), GCS (–21.47%), GLS (–17.95%), normal T1 (1,251 ± 5 ms) and ECV (30.1 ± 2.1%) for the left ventricular. **(Column 2)** An HCM patient showed patch LGE and GRS (59.03%), GCS (–15.31%), GLS (–5.61), increased native T1 (1331 ± 8.1 ms) and ECV (31.1 ± 3.2%) for the left ventricular. **(Column 3)** An AL patient of Mayo I showed patchy LGE and GRS (31.82%), GCS (–18.31%), GLS (–10.67), increased native T1 (1,324 ± 21 ms) and ECV (36.4 ± 3.1%) for the left ventricular. **(Column 4)** An AL patient of Mayo II showed slightly diffuse LGE at the myocardium and GRS (25.79%), GCS (–15.84%), GLS (–7.13%), increased native T1 (1,422 ± 3 ms) and ECV (38.9 ± 3.5%) for the left ventricular. **(Column 5)** An AL patient of Mayo III showed global extensive LGE at the myocardium and GRS (10.13%), GCS (–8,88%), GLS (–7.09%), increased native T1 (1,421 ± 57 ms) and ECV (57.0 ± 4.6%) for the left ventricular. 4CH, four-chamber; SA, short axis; LV, left ventricular; LGE, late gadolinium enhancement; HCM, hypertrophic cardiomyopathy patients; GRS, global radial strain; GCS, global circumferential strain; GLS, global longitudinal strain; ECV, extracellular volume.

Representative examples of LGE images and LV strain from healthy control, an HCM patient, and AL CA patients with different Mayo stages are in Figure 3. Healthy controls displayed no LGE. HCM patients and Mayo stage I/II AL CA patients could show no LGE or present atypical LGE, such as patchy or slightly diffuse LGE, in the septal mid-wall of the LV. Mayo stage IIIa/IIIb patients showed extensive LGE. The LV strain values of healthy controls, HCM patients, and different Mayo stage patients showed in Tables 1, 2 as a point spread diagram. HCM patients and AL CA patients showed impaired GLS (−16.8 ± 1.2 vs. −9.5 ± 1.8 vs. −11.2 ± 3.3%, all *P* < 0.001), respectively, compared to healthy controls. HCM patients showed the higher global circumferential strain (GCS) (−15.0 ± 3.2 vs. −21.2 ± 1.8%, *P* < 0.001) compared to healthy controls. Especially, Mayo stage I/II AL CA patients showed impaired LV GCS (−19.0 ± 3.6 vs. −15.0 ± 3.2%, *P* < 0.05) and LV GLS (−11.9 ± 3.0 vs. −9.5 ± 1.8%, *P* < 0.05) compared to HCM patients. Intra- and inter-observer reproducibility of myocardial deformation parameters (including GRS, GCS, and GLS) showed in Table 3.

**TABLE 3:** Intra-observer and inter-observer intraclass correlation coefficient of variabilities.

	LV GRS	LV GCS	LV GLS	Native T1	ECV
Intra	0.978	0.989	0.982	0.932	0.917
Inter	0.931	0.923	0.918	0.872	0.863

LV, left ventricle; GRS, global radial strain; GCS, global circumferential strain; GLS, global longitudinal strain; ECV, extracellular volume.

### Late gadolinium enhancement, native T1, and extracellular volume mapping

**FIGURE 4:**
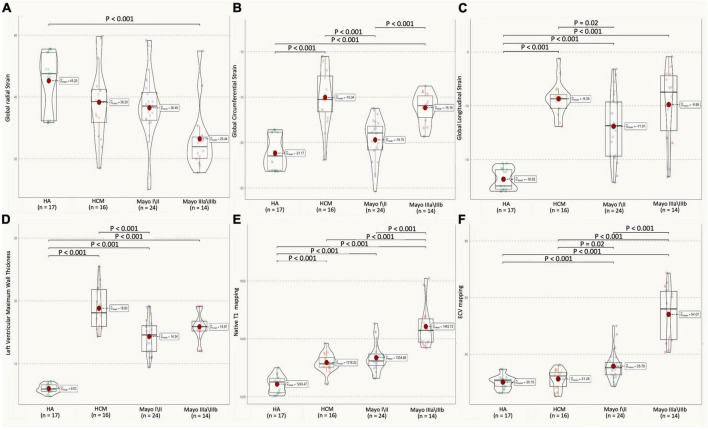
Multiparameter CMR (GRS, GCS, GLS of left ventricular, LVMT, native Tl, ECV values) in HA, HCM, AL amyloidosis patients with different Mayo stages. **(A)** AL CA patients showed a decrease in LV GRS strain (H: 45.2 ± 9.4%, HCM: 38.3 ± 12.0%, M I/II: 36.5 ± 10.9%, M III: 26.4 ± 10.7%; H vs. Mayo III, *P* < 0.001) as compared to healthy controls. **(B)** AL CA patients showed an increase in LV GCS strain (H: –21.2 ± 1.8%, HCM: –15.0 ± 3.2%, M I/II: –19.0 ± 3.6%, M III: –16.2 ± 1.7, HCM vs. M I/II, M I/II vs. M III, *P* = 0.02), as compared between them. **(C)** Mayo stage I/II AL patients showed an increase in the absolute value of LV GLS strain (H: –16.8 ± 1.2%, HCM: –9.5 ± 1.8%, M I/II: –11.9 ± 3.0%, M III: –9.9 ± 3.5, HCM vs. M I/II, *P* = 0.02), as compared to HCM. **(D)** HCM patients showed an increase in LVMWT (H: 6.1 ± 0.7 mm, HCM: 18.8 ± 3.3 mm, M I/II: 14.3 ± 2.9 mm, M III: 15.9 ± 2.3 mm, HCM vs. M I/II, *P* < 0.001), as compared to Mayo I/II AL patients. **(E)** AL patients showed an increase in native Tl mapping (H: 1,243.5 ± 29.85 ms, HCM: 1,318.2 ± 32.4 ms, M I/II: 1,334.9 ± 49.9 ms, M III: 1,442.7 ± 74.9 ms, H vs. M I/II, HCM vs. M III, Mayo I/II vs. Mayo III, *P* < 0.001, respectively), as compared between them. **(F)** AL CA patients showed an increase in ECV mapping (H: 30.1 ± 2.5%, HCM: 31.3 ± 3.4%, M I/II: 37.8 ± 5.7%, M III: 54.1 ± 9.9%, H vs. M I/II, Mayo I/II vs. Mayo III, *P* < 0.001, respectively, HCM vs. M I/II, *P* = 0.02), as compared between them. All CMR parameter values of AL patients and healthy control objects were shown as a point spread diagram; pairs with significant differences (*P* < 0.001) are connected with lines. CMR, cardiovascular magnetic resonance; HA, healthy control subjects; HCM, hypertrophic cardiomyopathy patients; M I/II, Mayo I/II AL amyloidosis patients; M III, Mayo IIIa/IIIb AL amyloidosis patients; LGE, late gadolinium GRS, global radial strain; GCS, global circumferential strain; GLS, global longitudinal strain; LVMT, left ventricular maximum wall thickness; ECV, extracellular volume.

Examples of short axis-and four-chamber LGE images, native T1, and ECV values from a healthy control subject, an HCM patient, and AL CA patients with different Mayo stages showed in Figure 3. The native T1 and ECV values of all AL CA patients and healthy controls showed in Figure 4 as a point spread diagram and Table 2. AL amyloidosis patients showed significantly higher native T1 values (1,374.6 ± 79.4 vs. 1,318.2 ± 32.4 vs. 1,243.5 ± 29.5, all *P* < 0.001) and ECV values (42.8 ± 11.5 vs. 31.3 ± 3.4 vs. 30.1 ± 2.5, *P* < 0.001) than HCM patients and health control subjects. Meanwhile, AL amyloidosis patients at Mayo stage IIIa/IIIb had significant higher native T1 values than patients at Mayo I/II stage or HCM patients (HCM: 1,318.2 ± 32.4 ms, M I/II: 1,334.9 ± 49.9 ms, M III: 1,442.7 ± 74.9 ms, HCM vs. M III, Mayo I/II vs. Mayo III, *P* < 0.001, respectively). ECV values were the lowest in HCM patients, median in Mayo I/II stage AL CA patients, and the highest in Mayo stage III patients (HCM: 31.3 ± 3.4%, M I/II: 37.8 ± 5.7%, M III: 54.1 ± 9.9%, HCM vs. M I/II, HCM vs. M III, Mayo I/II vs. Mayo III, *P* < 0.001, respectively). Reader reproducibility of native T1 and ECV was tested using the intraclass correlation coefficient (ICC). The results showed in Table 3.

### Association between cardiovascular magnetic resonance parameters and clinical stage

**FIGURE 5:**
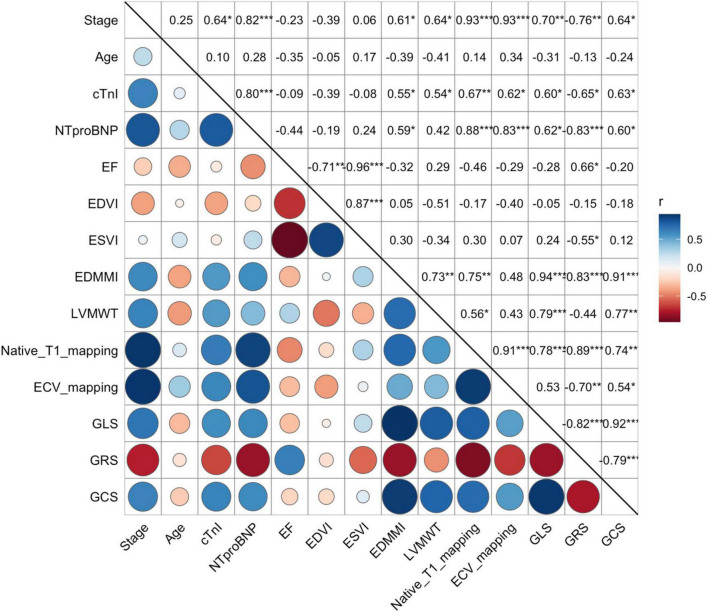
Correlation between the Mayo stage, biochemical markers (cTnI and NT-proBNP), and all CMR-related parameters, including EF, EDVI, ESVI, EDMMI, LVMWT, Native Tl mapping, ECV mapping, GLS, GRS, and GCS). Both native Tl mapping, ECV mapping, GLS, GRS, and GCS were strongly correlated to the Mayo stages (*r* = 0.93, 0.93, 0.70, 0.76, 0.64, respectively). However, EF, EDVI, and ESVI showed no significant correlation. Correlation between continuous variables was assessed using Pearson’s *r* correlation, such as native Tl, ECV mapping and strain values, etc., and with categorical variables using Spearman *P* correlation, such as Mayo stage. cTnI, Cardiac Troponin I; NT-proBNP, N-terminal pro-B-type natriuretic peptide; EF, left ventricle ejection fraction; EDVI, left ventricle end-diastolic volume index; ESVI, left ventricle end-systolic volume index; EDMMI, left ventricle end-diastolic maximum mass index; LVMWT, left ventricle maximum wall thickness; ECV, extracellular volume; GCS, global circumferential strain; GLS, global longitudinal strain; GRS, global radial strain. Symbols: *P*, Spearman correlation coefficient; *r*, Pearson correlation coefficient; blue, positive correlation; red, negative correlation; circle size, the degree of the correlation.

Table 2 and Figure 5 summarize the correlation of CMR parameters with disease classification, including the AL CA patients (Mayo stage I/II, Mayo stage IIIa/IIIb), and HCM patients. The LVMWT and MI of LV correlated significantly with the disease classification (*r* = 0.64 and 0.61, respectively, all *P* < 0.001). LV strain and native T1, as well as ECV, showed a significant correlation with disease classifications of patients (GRS, GCS, GLS, native T1, ECV, *r* = −0.76, 0.64, 0.70, 0.93, and 0.93, respectively, all *P* < 0.001). Compared to HCM patients, the CMR parameters (Native T1 mapping and ECV mapping) of Mayo stage III AL CA patients have significant differences (all *P* < 0.001). For Mayo stage I/II patients, the GLS absolute value (11.9 ± 3.0 vs. 9.5 ± 1.8, *P* < 0.001) and GCS absolute value (19.0 ± 3.6 vs. 15.0 ± 3.2, *P* = 0.02) were significantly higher than that of HCM patients. In addition, ECV values (37.8 ± 5.7 vs. 31.3 ± 3.4%, *P* < 0.0001) of Mayo stage I/II patients were higher than that of HCM patients.

### Multi-parameters cardiovascular magnetic resonance for identifying cardiac light-chain amyloidosis patients of Mayo stage I/II

**FIGURE 6:**
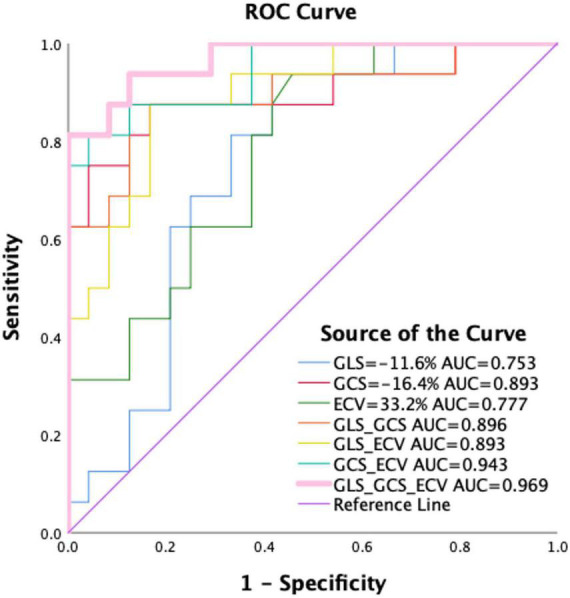
Receiver operating characteristics curve for GLS, GCS, and ECV mapping and pairwise parameter for differentiating between Mayo I/II AL amyloidosis and HCM patients. As shown, a GCS < –16.4%, GLS < –11.6%, or ECV > 33.2% could predict Mayo I/II amyloidosis from HCM patients. Among them, the combined diagnostic performance of GLS, GCS, and ECV mapping can well distinguish Mayo I/II AL amyloidosis patients from hypertrophic cardiomyopathy (AUC = 0.969, Youden index = 0.813). HCM, hypertrophic cardiomyopathy patients; ECV, extracellular volume; GLS, global longitudinal strain; GRS, global radial strain; AUC, area under the curve.

Tables 2, 4 and Figure 4 showed that LV MI, LVMWT, LV GLS, LV GCS, and ECV (all *P* < 0.001) have significant differences between Mayo stage I/II and HCM patients. Univariate binary logistic regression analysis showed that GCS global (HR = 2.097, 95% CI: 1.292–3.403, *P* = 0.003), GLS global (HR = 1.468, 95% CI: 1.078–1.998, *P* = 0.015) and ECV (HR = 0.727, 95% CI: 0.569–0.929, *P* = 0.011) could significantly discriminate for Mayo stage I/II and HCM patients dependently. Figure 6 and Table 5 show that a GCS < −16.4%, GLS < −11.6% or ECV > 33.2% could predict Mayo stage I/II amyloidosis (AUC = 0.893, 0.753, and 0.777, respectively; Youden index, *P* = 0.708, 0.521, and 0.48, respectively).

**TABLE 4:** Univariable and multivariable binary logistic regression for the diagnostic factors between Mayo stage I/II AL CA and HCM patients.

Characteristic	Univariate analysis		Multivariate analysis							
	HR (95% CI)	P	GLS + GCS		GLS + ECV		GCS + ECV		GLS + GCS + ECV	
			HR (95% CI)	*P*	HR (95% CI)	*P*	HR (95% CI)	*P*	HR (95% CI)	*P*
LVMWT	3.472 (0.665–18.132)	0.140								
LV GCS (%)	2.097 (1.292–3.403)	0.003	1.994 (1.213–3.280)	0.007			2.097 (1.263–3.482)	0.004	2.368 (1.160–4.832)	0.018
LV GLS (%)	1.468 (1.078–1.998)	0.015	1.156 (0.785–1.700)	0.462	0.571 (0.369–0.884)	0.012			2.802 (0.926–8.477)	0.068
Native T1 (ms)	0.990 (0.975–1.007)	0.246								
ECV (%)	0.727 (0.569–0.929)	0.011			2.094 (1.175–3.370)	0.012	0.657 (0.429–1.006)	0.053	0.360 (0.139–4.832)	0.035

All statistically significant prognostic factors between Mayo stage I/II AL CA patients and HCM control in univariate analysis were listed. All statistically significant variables in univariate analysis were put into the binary logistic regression predict model. MI, GLS, and native T1 were put in separate models with ECV because they have a strong correlation with each other (MI vs. GLS, GLS vs. native T1, MI vs. native T1, *r* and *P* = 0.730, 0.643, 0.605, respectively). AL CA, cardiac light-chain amyloidosis; HCM, hypertrophic cardiomyopathy patients; LVMWT, left ventricle maximum wall thickness; GLS, global longitudinal strain; HR, hazard ratio; CI, confidence interval.

**TABLE 5:** The diagnostic value of cardiovascular magnetic resonance to differentiate between Mayo stage I/II AL CA and HCM patients.

Multi-parameters of CMR for differentiating between Mayo stage I/II AL CA patients and HCM subjects					
	AUC	Cut-off	Youden index	Sensitivity	Specificity
GCS (%)	0.893	−16.4	0.708	0.875	0.833
GLS (%)	0.753	−11.6	0.521	0.938	0.583
ECV (%)	0.777	33.2	0.48	0.938	0.542
GLS + GCS	0.896		0.75	0.875	0.875
GLS + ECV	0.893		0.708	0.875	0.844
GCS + ECV	0.943		0.771	0.813	0.958
GLS + GCS + ECV	0.969		0.813	0.813	1

AL CA, cardiac light-chain amyloidosis; HCM, hypertrophic cardiomyopathy patients; GCS, global circumferential strain; GLS, global longitudinal strain; ECV, extracellular volume.

Multivariate binary logistic regression analyses GLS, GCS, and ECV. GCS, GLS, and ECV were put in pairs and combinations of three. In the GLS + GCS model, GCS (1.994, 95% CI: 1.213–3.280, *P* = 0.007) was the only significant variable. In the GLS + ECV model, GLS (0.571, 95% CI: 0.369–0.884, *P* = 0.012) and ECV (2.094, 95% CI: 1.175–3.370, *P* = 0.012) were the significant variable. In the GCS + ECV model, GCS (2.097, 95% CI: 1.263–3.482, *P* = 0.004) and ECV (0.657, 95% CI: 0.429–1.006, *P* = 0.053) were the significant variable. The combinations of the three showed the best performance for identifying AL CA patients of Mayo stage I/II from HCM patients (AUC: 0.969, Youden index: 0.813). GCS (2.368, 95% CI: 1.160–4.832, *P* = 0.018) and ECV (0.360, 95% CI: 0.139–4.832, *P* = 0.035) were the significant variable.

## Discussion

As demonstrated in selected patient populations with AL CA, we found that GCS and GLS in combination with ECV could effectively discriminate Mayo stage I/II patients from HCM patients. First, we comprehensively assessed the differences in CINE, LGE, T1 mapping value, ECV value, and LV myocardial strain between the myocardium of AL CA patients with Mayo stage I/II, IIIa/IIIb stages, HCM patients, and healthy controls. Second, GCS, GLS, and ECV correlate highly with clinical classification. Furthermore, Mayo stage I/II AL CA patients, who had atypical LGE, GCS, GLS, and ECV can be used as independent diagnostic factors compared to HCM patients. To the best of our knowledge, this is the first study to assess the diagnostic value of multi-parameter CMR concurrently with detecting light-chain amyloidosis with earlier stages. This important finding may provide a basis for the development of a novel tool for the diagnosis of early-stage AL CA.

Due to the increased interstitial volume, extracellular gadolinium-containing contrast media (CM), which is commonly used in CMR, exhibits a correspondingly increased volume of distribution in the heart with amyloidosis (25, 26). In the classic or Mayo stage III AL CA patients, CM accumulates in all infiltrated myocardial segments. The LGE method is particularly effective at visualizing the extensive signals of CM. Based upon a meta-analysis of five studies (*n* = 257) it was concluded that the LGE method has an estimated sensitivity and specificity of 85 and 92%, respectively, for the diagnosis of cardiac amyloidosis (7). In the workup of cardiac amyloidosis, CMR has developed into a versatile tool with superior tissue characterization, high-resolution imaging, and precise cardiac assessment. The presence of these features allows us to confirm cardiac involvement, differentiate AL CA from other cardiomyopathies, and assess the morphological and functional status of the heart. Takeda et al. highlighted the capability of CMR in differentiating AL CA (*n* = 6) from hypertrophic cardiomyopathies (HCM, *n* = 9) and hypertensive heart disease (HHD, *n* = 11). In several subsequent studies, co-localized endocardial biopsy confirmed the high diagnostic accuracy of LGE patterns in AL CA; however, in patients with early-stage AL CA, mild amyloid deposition in the myocardium may contribute to atypical features of LGE (4, 27).

In AL CA patients, subendocardial delayed enhancement occurs primarily in the early stages, while transmural delayed enhancement occurs mostly in the middle and late stages (7). Hence, patients with the early-stage or Mayo stage I/II AL CA always had preclinical myocardial damage, which is not diffuse and homogeneous as typical LGE patterns of AL CA patients (28). For atypical LGE patterns, it is challenging to indicate that amyloidosis has occurred (29, 30). In addition, the symptoms of AL CA patients in the early stages are similar to those of HCM patients. The typical manifestations of AL CA are diffuse thickening of the LV wall, mainly with thickening of the ventricular septum, while hypertrophic cardiomyopathy is mostly asymmetric thickening. In addition to limitations in diastolic and large function, typical LGE delayed enhancement can be used for differential diagnosis (31–34). Still, in patients with early myocardial amyloidosis without visible thickening of the ventricular wall and hypertrophic heart patients without significant fibrosis, it is difficult to distinguish between the two based on morphology and function (27). And conventional CMR images and electrocardiograms are often overlapping and confusing, making it difficult to diagnose early-stage AL CA patients precisely. In addition, hypertension was not listed as an exclusion criterion in our study. It is acknowledged that hypertension can affect wall thickness even if the hypertension is well controlled (35). Nevertheless, due to the high prevalence of hypertension, this is a real-world situation. Meanwhile, a frequently raised concern is the administration of gadolinium-containing CM to patients with impaired renal function. Hence, contrast-free multi-parameter CMR emerges as a non-invasive and comprehensive tool to detect early-stage AL CA from HCM patients and monitor its progression have dominated the current research efforts related to CA (4).

Amyloid deposition in the heart would impair cardiac function. The LVEF has been the cornerstone of measuring cardiac function. LVEF, however, is not able to detect slight diastolic dysfunction in the early-stage (Mayo stage I/II) AL CA patients (36). Our results also indicated no difference in EF between HCM patients and early-stage AL CA patients. The LV strain has been proven to be a sensitive and robust indicator of cardiac dysfunction. CMR Strain analysis allows a more direct assessment of the left ventricle function than conventional LVEF (28). GLS, GCS and GRS are caused by the involvement of subendocardial fibers, the involvement of subepicardial fibers, and transmural involvement, respectively. In this study, the strain capacity of the different groups was analyzed using magnetic resonance tissue tracking technology. The results showed that, compared to healthy controls, the myocardial strain rates of LV GRS, GCS, and GLS in patients with myocardial amyloidosis and the hypertrophic heart group were decreased to varying degrees. We and others (12, 37) demonstrated that LV GLS is an independent predictor for AL CA when differentiating AL CA patients from HCM patients. There was no significant difference in the GLS strain capacity between the HCM group and the Mayo III group (*P* > 0.05); however, the GLS of the HCM group is higher than that of Mayo I/II groups (−9.5 ± 1.8 vs. −11.9 ± 3.0%, *P* < 0.05). In both HCM and Mayo III groups, the significant decrease in GLS, compared to that of healthy controls, was due to local or diffuse myocardial fibrosis, amyloid deposition, and secondary coronary microcirculation dysfunction. The pathological changes can result in ischemia of corresponding myocardial segments, reduced myocardial contractility and compliance, and abnormal myocardial movement (38). For Mayo stage I/II patients, even though the extracellular matrix of the myocardium had not been invaded by the amyloid protein and the LV function was generally normal in the early-stage AL CA, the thickening of the myocardium also reduced myocardial compliance. Thus, in this study, their GLS value is different from HCM patients, which makes it more sensitive to identify the early-stage of AL CA. Similar to Wan et al. (39) study, we found that GCS has a statistically significant difference between different Mayo staging groups (*P* < 0.001). The study found that HCM patients, Mayo stage I/II patients, and Mayo stage IIIa/IIIb patients had different degrees of thickening of the ventricular wall, reduced cardiac function, and diastolic function limitations. Given that GCS and LVMWT have a strong correlation (*r* = 0.77, *P* < 0.001), we believe that when the myocardial thickness is deformed, GCS can more accurately reflect the changes in myocardial function. Therefore, GCS can provide a reference value for the differential diagnosis of HCM and Mayo staging. The GLS and GCS were significantly correlated with the Mayo stage (*r* = 0.70, 0.64). The best cut-off value of GLS is −11.6%, and GCS is −16.4%, which may be a diagnostic factor for Mayo stage I/II AL CA patients from HCM patients.

Quantitative T1 mapping has also demonstrated great potential in the detection, differentiation, and stratification of AL CA. Previous studies have demonstrated the diagnostic value of native T1 and ECV for mortality using a 1.5T or 3.0T scanner with a MOLLI sequence (10, 40), but these studies did not include patients with early-stage AL CA. And HCM is characterized by an increase in the ECV as the earliest pathological change, which is similar to early-stage AL CA patients. Here, we included HCM patients in the group setting to make a comprehensive study. The pairwise comparison between the Mayo stage I/II/III groups, HCM patients, and the healthy control group was used. The T1 mapping and ECV values of HCM and AL CA were higher than those of the normal control group (1243.5 ± 29.5 vs. 1318.2 ± 32.4 vs. 1374.6 ± 74.9 ms, both *P* < 0.001; 30.1 ± 2.5 vs. 31.3 ± 3.4 vs. 42.8 ± 11.5%, *P* < 0.001, respectively). This study had similar results, which is consistent with other studies (10, 41). And the ECV values of Mayo stage I/II AL CA were significantly higher than those of HCM, and the difference was statistically significant (31.3 ± 3.4 vs. 37.8 ± 5.7%, *P* < 0.05). T1 mapping and ECV significantly correlated with the Mayo stage (*r* = 0.93, 0.93). Based on the findings of this study, T1 mapping values can be used to differentiate Mayo staging from healthy people. For patients with early-stage myocardial amyloidosis (Mayo stage I/II) who did not show LGE or atypical patchy enhancement of the LV muscle wall or atypical subendocardial enhancement, both native T1 mapping and ECV values were increased, indicating that T1 mapping and ECV value are more sensitive than LGE in detecting early myocardial involvement in patients with AL. However, there was no statistically significant difference between the Mayo stage I/II groups and the HCM group. This may be due to focal fibrosis in both HCM and early-stage AL CA patients resulting in similar T1 mapping values. Importantly, the ECV value of the Mayo I/II group was higher than that of the HCM group and significantly lower than the Mayo IIIa/IIIb group (HCM: 31.3 ± 3.4% vs. Mayo I/II: 37.8 ± 5.7% vs. Mayo IIIa/IIIb: 54.1 ± 9.9%, *P* < 0.001). Our results showed that the best cut-off value of ECV is 33.2%. This value is lower than the value of Lin et al. study (ECV > 44%) (10). The area under the ROC curve of ECV was 0.777 (Youden index = 0.48). The difference between Mayo stage patients may partially explain the discrepancy. The relatively lower AUC of ECV could be explained by the absence of extensive fibrosis in Mayo I/II patients who just had amyloid deposits. Extracellular spaces are expanded by fibrosis, not just by amyloid deposits, as demonstrated by Pucci et al. (42).

N-terminal pro-B-type natriuretic peptide and cTnI have established biomarkers for evaluating the severity of myocardial involvement and are associated with patient prognosis (43). The elevation of BNP correlates closely with the accumulation of myocardial amyloid. It can be increased in the early period of amyloidosis and before the onset of abnormal electrocardiogram, including in asymptomatic patients (44). In this study, 2 of 38 patients with Mayo stage I/II had elevated NT-proBNP and cTnI. These patients have typical or atypical CMR LGE, elevated T1 mapping, and ECV values, and diminished GCS and GLS. NT-proBNP was significantly elevated in all Mayo IIIa/IIIb patients, NT-proBNP was significantly elevated, whereas LGE showed typical enhancement. Moreover, T1 mapping and ECV values were enriched significantly while GLS and GCS were considerably reduced in these patients. These results supported that GCS, GLS, and ECV could detect early myocardial involvement correlates with NT-proBNP, which indicated the value of GCS, GLS, and ECV for evaluating the severity of myocardial amyloidosis. In this study, the combination of GLS, GCS, and ECV yielded excellent diagnostic performance in differentiating patients with early myocardial involvement (AUC = 0.969, sensitivity = 0.813, specificity = 1, respectively) from HCM patients.

There are a few inherent drawbacks of this study. The main limitation is that this is a single-center study. Despite the success of the internal validation using clinical parameters and biomarkers to verify the performance of the multi-parameters CMR in recognizing early-stage AL CA, a multi-center prospective study with different vendors is still necessary to validate our findings. Second, some high prevalence conditions, such as hypertension, diabetes, etc., were not listed as exclusion criteria in our study. We acknowledged that hypertension can influence the wall thickness (35). Compared to the HCM patients, however, they have different LGE patterns, GCS, GLS, and ECV values. Also, the classification of AL CA patients is based on biochemical blood parameters in the clinical guidelines (16, 17). The rare cardiac transthyretin amyloidosis (ATTR CA) type was not included, and those without contrast CMR scans were excluded. More male patients were included, and patient selection bias may exist. Third, echocardiography retains its superiority in clinical practices, but it is limited by productivity and operator independence. Furthermore, it is necessary to verify whether the diagnostic value can be interchangeable between CMR and echocardiography. Fourth, in our study, the sample size of the Mayo-stage AL CA patients is small. Here we set Mayo stage I and Mayo stage II AL CA patients together as early-stage AL CA patients. The confirmation of early myocardial amyloidosis requires a myocardial biopsy to differentiate between amyloid deposition and myocardial fibrosis. However, due to the invasiveness of myocardial biopsy, the lack of pathological evidence for cardiac involvement is rarely presented in studies related to AL CA.

## Conclusion

Left ventricular GCS and LV GLS parameters have good diagnostic values at different stages of myocardial amyloidosis. In early-stage AL CA patients, who have atypical LGE, GCS, GLS, and ECV are highly correlated with their clinical classification and have been altered when compared with HCM patients and typical amyloidosis. The combination of GCS, GLS, and ECV could accurately differentiate early-stage AL CA from healthy controls or HCM patients. It could be as a novel approach to monitoring the CMR characteristics of early-stage AL CA patients with multiple follow-ups.

## Data availability statement

The original contributions presented in this study are included in the article/supplementary material, further inquiries can be directed to the corresponding author.

## Ethics statement

All subjects have consented to participate in this study. This research was approved by the Institutional Ethics Committee for Human Research at the People’s Hospital of Ningxia Hui Autonomous Region (Yinchuan, China). The patients/participants provided their written informed consent to participate in this study.

## Author contributions

XY, RW, and ZZ performed patient scanning data analyses. XY and FW wrote the main draft of the manuscript. YY and XR collected basic information about patients. LY, XW, and YW provided clinical baseline information and pathological analysis. XY, RW, and QC were involved in interpreting the results. All authors read and approved the final manuscript.

## Abbreviations

MR: magnetic resonance
2D: two-dimensional
AL CA: cardiac light-chain amyloidosis
AL: light-chain
ANOVA: one-way analysis of variance
bSSFP: balanced steady-state free precession
CI: confidence intervals
CMR: cardiovascular magnetic resonance
cTnI: Cardiac Troponin I
ECG: electrocardiogram
ECV: extracellular volume fraction
FA: flip angle
LGE: late gadolinium enhancement
LV: left ventricle/left ventricular
LVEDV: left ventricle end-diastolic volume index
LVEF: left ventricular ejection fraction
MOLLI: modified look-locker inversion-recovery
NT-proBNP: N-terminal pro-B-type natriuretic peptide
PSIR: phase-sensitive inversion-recovery
TE: echo time
TR: repetition time
NYHA: New York Heart Association.
